# Molecular Guardians of Oocyte Maturation: A Systematic Review on TUBB8, KIF11, and CKAP5 in IVF Outcomes

**DOI:** 10.3390/ijms26136390

**Published:** 2025-07-02

**Authors:** Charalampos Voros, Ioakeim Sapantzoglou, Diamantis Athanasiou, Antonia Varthaliti, Despoina Mavrogianni, Kyriakos Bananis, Antonia Athanasiou, Aikaterini Athanasiou, Georgios Papadimas, Athanasios Gkirgkinoudis, Ioannis Papapanagiotou, Kyriaki Migklis, Dimitrios Vaitsis, Aristotelis-Marios Koulakmanidis, Dimitris Mazis Kourakos, Sofia Ivanidou, Maria Anastasia Daskalaki, Marianna Theodora, Panagiotis Antsaklis, Dimitrios Loutradis, Georgios Daskalakis

**Affiliations:** 1 Department of Obstetrics and Gynecology, ‘Alexandra’ General Hospital, National and Kapodistrian University of Athens, 11528 Athens, Greece; kimsap1990@hotmail.com (I.S.); antonia.varthaliti@hotmail.com (A.V.); depy.mavrogianni@yahoo.com (D.M.); aristoteliskoulak@gmail.com (A.-M.K.); md181341@students.euc.ac.cy (M.A.D.); martheodr@gmail.com (M.T.); panosant@gmail.com (P.A.); gdaskalakis@yahoo.com (G.D.); 2 IVF Athens Reproduction Center V. Athanasiou, 15123 Maroussi, Greece; diamathan16@gmail.com (D.A.); antoathan16@gmail.com (A.A.); diamathan17@gmail.com (A.A.); 3 King’s College Hospitals NHS Foundation Trust, London SE5 9RS, UK; kyriakos.bananis@nhs.net; 4 Athens Medical School, National and Kapodistrian University of Athens, 15772 Athens, Greece; dr.georgepapadimas@gmail.com (G.P.); gpapamd@hotmail.com (I.P.); kyriaki.migklis@gmail.com (K.M.); vaitsisdim@gmail.com (D.V.); mazisdimitris@gmail.com (D.M.K.); info@ivanidou.gr (S.I.); loutradi@otenet.gr (D.L.); 5 Assisted Reproduction Unit, Fertility Institute, Paster 15, 11528 Athens, Greece

**Keywords:** *TUBB8*, *KIF11*, *CKAP5*, oocyte maturation arrest, spindle assembly, early embryonic arrest, IVF failure, meiotic spindle, cytoskeleton, microtubule dynamics, female infertility, genetic variants, assisted reproductive technology

## Abstract

The efficacy of in vitro fertilization (IVF) is significantly hindered by early embryonic developmental failure and oocyte maturation arrest. Recent findings in reproductive genetics have identified several oocyte-specific genes—*TUBB8*, *KIF11*, and *CKAP5*—as essential regulators of meiotic spindle formation and cytoskeletal dynamics. Mutations in these genes can lead to significant meiotic defects, fertilization failure, and embryo arrest. The links between genotype and phenotype, along with the underlying biological mechanisms, remain inadequately characterized despite the increasing number of identified variations. This systematic review was conducted in accordance with PRISMA 2020 guidelines. Relevant papers were retrieved from the PubMed and Embase databases using combinations of the keywords “*TUBB8*,” “*KIF11*,” “*CKAP5*,” “oocyte maturation arrest,” “embryonic arrest,” and “IVF failure.” Studies were included if they contained clinical, genomic, and functional data on *TUBB8*, *KIF11*, or *CKAP5* mutations in women undergoing IVF. Molecular data, including gene variant classifications, inheritance models, in vitro tests (such as microtubule network analysis in HeLa cells), and assisted reproductive technology (ART) outcomes, were obtained. Eighteen trials including 35 women with primary infertility were included. Over fifty different variants were identified, the majority of which can be attributed to *TUBB8* mutations. *TUBB8* disrupted α/β-tubulin heterodimer assembly due to homozygous missense mutations, hence hindering meiotic spindle formation and leading to early embryo fragmentation or the creation of many pronuclei and cleavage failure. *KIF11* mutations resulted in spindle disorganization and chromosomal misalignment via disrupting tubulin acetylation and microtubule transport. Mutations in *CKAP5* impaired bipolar spindle assembly and microtubule stabilization. In vitro validation studies showed cytoskeletal disturbances, protein instability, and dominant negative effects in transfected animals. Donor egg IVF was the sole effective treatment; however, no viable pregnancies were documented in patients with pathogenic mutations of *TUBB8* or *KIF11*. *TUBB8*, *KIF11*, and *CKAP5* are essential for safeguarding oocyte meiotic competence and early embryonic development at the molecular level. Genetic differences in these genes disrupt microtubule dynamics and spindle assembly, resulting in various aspects of oocyte maturation and fertilization. Functional validation underscores the necessity of routine genetic screening for women experiencing unresolved IVF failure, as it substantiates their causal role in infertility. Future therapeutic avenues in ART may be enhanced by tailored counseling and innovative rescue methodologies like as gene therapy.

## 1. Introduction

Oocyte maturation and early embryonic development are intricate processes requiring precise coordination of cytoskeletal dynamics, spindle formation, chromosomal segregation, and cell cycle progression [1]. The meiotic spindle, which is a microtubule-based acentrosomal structure, is at center stage in the process of assuring proper chromosome segregation in meiosis I and II [2]. Human oocytes form and maintain the meiotic spindle with specialized microtubule organizing centers (MTOCs) and precisely regulated molecular motors, in contrast to somatic cells that depend on centrosomes [3]. The characteristics of primary female infertility unresponsive to conventional ART frequently manifest as oocyte maturation arrest, fertilization failure, and cessation of early embryonic development due to genetic anomalies in this meticulously regulated system [4].

Recent advancements in genomics and functional molecular biology have identified a group of genes whose protein products serve as essential molecular regulators, or “guardians,” of oocyte competency. *TUBB8*, *KIF11*, and cytoskeleton-associated protein 5 *(CKAP5)* are notable for their critical functions in microtubule structure and spindle dynamics [5,6,7]. Mutations in these genes have been demonstrated to impede meiotic progression through dominant negative or loss-of-function mechanisms, resulting in a range of phenotypes such as germinal vesicle (GV) arrest, metaphase I (MI) arrest, abnormal polar body extrusion, multi-pronuclei formation, cleavage failure, and ultimately embryo fragmentation or implantation failure [5,6,7].

The β8 isoform of tubulin, a β-tubulin particular to primates that joins with α-tubulin to form the α/β heterodimer—the fundamental unit of microtubules—is encoded by *TUBB8*. *TUBB8*, mostly expressed in oocytes and early embryos, is the principal β-tubulin isoform involved in meiotic spindle formation [8]. The storage of maternal mRNA and the regulation of translation during oocyte development precisely govern *TUBB8* expression. β-tubulin functionally regulates microtubule stability, interactions with motor proteins, and resistance to depolymerization by several post-translational modifications (PTMs), such as acetylation, detyrosination, polyglutamylation, and glycylation [9]. Variants in TUBB8, especially missense mutations adjacent to the GTP-binding domain or dimer interface, impair tubulin folding (facilitated by the tubulin-specific folding pathway involving prefoldin, *CCT/TRiC*, and tubulin co-factors A–E), inhibit heterodimer formation, and disrupt microtubule polymerization dynamics [7]. These mutations disrupt the microtubule lattice and inhibit the incorporation of tubulin into stable spindle fibers, according to structural models. Importantly, aberrant TUBB8 disrupts spindle pole integrity and kinetochore– microtubule attachments, hence activating the SAC and halting the cell in meiosis I [10]. Oocytes exhibit an absence of homologous chromosomal segregation, leading to the production of either no or inadequate polar bodies, typically culminating in embryonic cleavage arrest or fertilization failure. Additionally, defective tubulin networks exacerbate spindle abnormalities, potentially hindering the recruitment of crucial regulators like as *TPX2*, *PLK1*, and Aurora kinase A (*AURKA*) [11].

*KIF11* (Eg5), a member of the kinesin-5 family of ATP-dependent motor proteins, traverses microtubules towards the plus end, generating the forces required to separate spindle poles and maintain spindle bipolarity [12]. *KIF11* is crucial for the organization of the meiosis I-specific bipolar structure of acentrosomal microtubules in oocytes. *KIF11* mechanistically produces outward sliding forces that separate microtubule-organizing centers by crosslinking antiparallel microtubules [13]. To maintain spindle pole integrity, it collaborates with other spindle bipolarizing components such as *HURP*, the dynein-dynactin complex, and *NuMA*. At Thr927, phosphorylation unique to *CDK1*/cyclin B1, *TPX2*, and Eg5 meticulously regulates *KIF11* activity [14]. The disruption of *KIF11* function results in the formation of monopolar or multipolar spindles, leading to chromosomal misalignment, activation of the SAC, and cessation of meiosis. Moreover, regulating microtubule acetylation through histone deacetylase 6 (*HDAC6*) activity modifies spindle rigidity and microtubule dynamics. The decline in oocyte quality is further exacerbated by a decrease in KIF11 activity, which has been linked to increased levels of reactive oxygen species (ROS) and ooplasmic damage.

*CKAP5*, sometimes referred to as ch-TOG or *XMAP215*, is crucial for spindle growth in the absence of centrioles and encodes a member of the microtubule polymerase family. *CKAP5* is localized in mammalian oocytes to a newly identified, transient structure composed of *CKAP5*, *TACC3*, *CCP110*, and *DISC1*, known as the huoMTOC [15]. This non-centrosomal microtubule-organizing center migrates to chromosomes during nuclear envelope breakdown, nucleating and organizing spindle microtubules to initiate bipolar spindle assembly. *CKAP5* interacts with tubulin dimers, facilitating their incorporation at the growing ends of microtubules. By interacting with proteins such as *CLASP1*, *TPX2*, and *KNL1*, it stabilizes kinetochore fibers and prevents microtubule catastrophe. Loss-of-function mutations in *CKAP5* alter spindle bipolarity and compromise microtubule nucleation, resulting in asymmetrical spindles, chromosomal missegregation, and the failure of polar body extrusion [7]. Additionally, *CKAP5* engages with mitotic kinases such as Aurora A and *PLK4* to facilitate the phosphorylation-mediated regulation of microtubule growth rates and microtubule-organizing center stability. Oocyte fragmentation, chromatin dispersion, and the formation of multipolar spindles have all been associated with problems in this system [16]. The oocyte meiotic spindle serves as a dynamic convergence of several signaling pathways and structural regulators, ensuring accurate chromosomal segregation and developmental capability [17]. Under sufficient strain, the SAC, a regulatory mechanism that inhibits the initiation of anaphase until all kinetochores are correctly attached to spindle microtubules, underscores the importance of this process [18]. Key *SAC* mediators are *MAD2*, *BUB1*, and *MPS1*, which, in the presence of unattached kinetochores, inhibit the function of the anaphase-promoting complex/cyclosome (APC/C).

Simultaneously with this checkpoint mechanism, the Aurora kinase family—particularly *AURKA* and *AURKB*—is crucial for regulating acentrosomal spindle pole dynamics and chromatin condensation [19]. AURKB ensures accurate kinetochore–microtubule attachments and rectifies chromosomal alignment, whereas *AURKA* is localized to microtubule-organizing centers and is essential for the formation of bipolar spindles [16]. Their effects are synchronized with the polo-like kinase 1 (*PLK1*) and *TPX2* axis, which regulates homologous chromosomal biorientation, kinetochore tension, and spindle formation during meiosis I [20,21].

Post-translational modifications of tubulin—such as acetylation, detyrosination, and polyglutamylation—that influence microtubule stability and interactions with motor proteins, including kinesins and dynein, contribute to this regulatory network [22]. These alterations delineate microtubule dynamic behavior and enhance the mechanical resilience of the meiotic spindle. Mitochondrial integrity and the control of ROS are becoming recognized as crucial elements in spindle activity. Excess reactive oxygen species can impair spindle microtubules and hinder cell cycle advancement, whereas mitochondrial ATP production facilitates the energy-demanding activities of motor proteins [23].

The many interactions among pathogenic mutations in *TUBB8*, *KIF11*, and *CKAP5* delineate how mutant *TUBB8* proteins impair spindle organization and hinder microtubule polymerization. *CKAP5* mutations destabilize microtubule-organizing center-driven microtubule nucleation, whereas *KIF11* deficiencies impair bipolar spindle development and tubulin acetylation. Each of these components induces checkpoint activation, meiotic arrest, and subsequent developmental incompetence. Any molecular alteration in this network might profoundly impact oocyte survival and embryonic development due to the oocyte’s specific dependence on acentrosomal spindle formation and temporally regulated maternal mRNA translation.

This systematic review aims to provide a comprehensive molecular and clinical synthesis of current knowledge regarding TUBB8, KIF11, and CKAP5 mutations in women undergoing IVF treatment. We aim to delineate the essential molecular pathophysiology of oocyte maturation arrest, which may subsequently lead to early embryonic developmental failure, by examining the genomic architecture, mutational spectra, functional implications, and pathway-level anomalies associated with these genes. This study seeks to find critical diagnostic indications within the realm of reproductive genetics and explore prospective avenues for precision medicine and targeted therapy.

## 2. Materials and Methods

### 2.1. Protocol and Registration

This systematic review was performed in accordance with the 2020 standards of the Preferred Reporting Items for Systematic Reviews and Meta-Analyses (PRISMA). The protocol was registered in the International Prospective Register of Systematic Reviews (PROSPERO) with ID CRD420251051343.

### 2.2. Search Methodologies

A comprehensive literature review was undertaken within the context of IVF or ICSI to identify all relevant studies investigating mutations in the *TUBB8*, *KIF11*, and *CKAP5* genes and their association with oocyte maturation arrest, spindle development issues, or early embryonic developmental failure. The search, spanning from January 2010 to April 2025, was conducted across four electronic databases: PubMed, Scopus, Web of Science, and Embase.

Medical Subject Headings (MeSHs) and free-text keywords included “*TUBB8*,” “*KIF11*,” “*CKAP5*,” “oocyte maturation,” “spindle defects,” “meiosis arrest,” “microtubule dynamics,” “embryo cleavage failure,” and “IVF failure” as search terms. The phrases were appropriately joined using Boolean operators (AND/OR).

Filters were applied to restrict the results to papers in English, those with full-text access, and research involving humans. A total of 467 papers were initially acquired: 142 from PubMed, 127 from Scopus, 95 from Web of Science, and 103 from Embase. Table 1 encompasses the complete search methodology and retrieval data.

Table 1 compiles the results of the systematic search methodology employed across four primary scientific databases to identify pertinent research about polymorphisms in the *TUBB8*, *KIF11*, and *CKAP5* genes and their association with oocyte maturation anomalies and IVF results. The search encompassed specific terms and Boolean operators, conducted from January 2010 to April 2025. Filters were applied to restrict the output to English-language human research that included accessible full texts.

### 2.3. Criteria for Eligibility

The PICOS (Population, Intervention, Comparator, Outcomes, Study Design) framework facilitated the a priori establishment of inclusion and exclusion criteria for this systematic review, ensuring the targeted acquisition of clinically and molecularly pertinent research. Within the context of assisted reproduction, pertinent study was mandated to investigate the role of genetic polymorphisms in TUBB8, KIF11, or CKAP5 and their association with oocyte maturation inadequacies, meiotic spindle anomalies, fertilization failures, or early embryonic developmental arrest.

Eligible study categories were original peer-reviewed articles such as case reports, case series, cohort studies, and experimental investigations involving human patients or functionally relevant cellular or animal models directly applicable to human infertility. We excluded reviews, conference abstracts, editorials, and duplicate publications.

To be included, studies must report data from women undergoing IVF or ICSI who have specific characteristics such as GV arrest, MI or metaphase II (MII) arrest, fertilization failure, cleavage failure, or embryonic fragmentation. Studies pertaining to male infertility or related illnesses were excluded.

Only studies explicitly examining mutations or dysregulation of the TUBB8, KIF11, or CKAP5 genes were included to ensure relevance to the molecular focus of this review. Studies without gene-level data or those restricted to general spindle or oocyte abnormalities without molecular characterization were excluded. Studies examining other genetic loci without concomitant reporting of *TUBB8*/*KIF11*/*CKAP5* results were also excluded.

Suitable research, categorized by sample type, must encompass human-derived biological materials such as oocytes, early embryos, granulosa or cumulus cells, or peripheral blood samples utilized for genetic analysis. Studies relying solely on non-human models (e.g., zebrafish or Drosophila) without human validation or extrapolation were excluded unless they provided mechanistic evidence explicitly linked to human orthologs.

Studies were required to present results from in vitro or in silico pathogenicity assessments, the identification of specific gene variants (via sequencing or molecular diagnostics), a connection with meiotic anomalies, spindle morphology, or clinical IVF outcomes at the molecular and/or phenotypic level. We excluded studies that lacked functional, structural, or phenotypic validation of alterations.

Ultimately, only English-language, readily accessible, full-text publications were considered eligible. To ensure repeatability and rigorous assessment of the methodologies, non-English publications, articles lacking full texts, and unpublished data were excluded from this review. Table 2 provides a comprehensive summary of the inclusion and exclusion criteria across all relevant domains.

Table 2 outlines the predetermined eligibility criteria used for selecting studies to be incorporated into the systematic review. The criteria were based on study design, target population, genetic focus, sample type, outcome relevance, and language limitations. Only original papers presenting human data with molecular or functional analyses of TUBB8, KIF11, or CKAP5 gene variants were deemed eligible.

### 2.4. Selection of Studies

In accordance with the PRISMA 2020 guidelines, the study selection process for this systematic review was meticulous and multi-phased to ensure transparency, reproducibility, and reduction of selection bias. Initially, all citations obtained from the electronic databases—PubMed, Scopus, Web of Science, and Embase—were exported into Zotero (v6.0), an open-source reference management software. Zotero’s integrated deduplication tool facilitated the identification and removal of duplicate data, followed by manual verification to ensure no extraneous entries remained. The generated collection was regarded as the master corpus for screening.

The initial phase of screening involved reviewing titles and abstracts for each deduplicated submission. Two blinded reviewers separately conducted this technique. At this stage, review papers, conference abstracts, editorials, and non-original studies were rejected if they utilized non-human models not directly pertinent to human oocyte development, or if they did not maintain a clear emphasis on the genetic or molecular analysis of TUBB8, KIF11, or CKAP5. Articles not written in English or lacking readily accessible entire texts were excluded. All screening decisions were documented using Rayyan QCRI, a web-based platform designed to provide blinded dual screening in systematic reviews. Any concerns encountered during this phase were resolved through candid discussion; in instances of persistent disagreement, a third senior reviewer served as an impartial arbitrator.

Articles deemed potentially suitable following title and abstract screening were subsequently acquired in full and meticulously reviewed. During this phase, each article was independently assessed by the same two reviewers according to the predetermined inclusion and exclusion criteria outlined in Section 2. Three studies that did not include original genetic or molecular data pertaining to *TUBB8*, *KIF11*, or *CKAP5*, failed to deliver pertinent oocyte or embryo-level phenotypic characterization, or did not report functional, structural, or clinical reproductive outcomes relevant to IVF or ICSI, were excluded at this stage. Additionally, studies utilizing solely animal models without verified human correlation were excluded from the final study.

**Figure 1:**
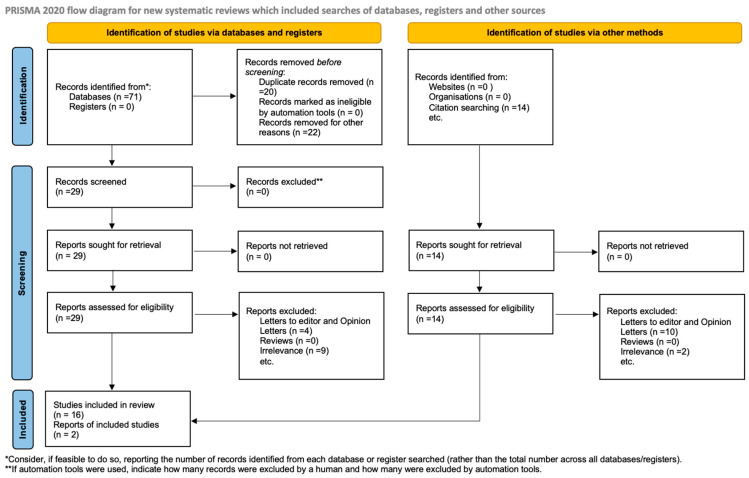
PRISMA 2020 flow diagram of the study selection process [24].

The PRISMA 2020 flow diagram (Figure 1) illustrates the comprehensive process of study selection; it documents the quantity of records retrieved from each database, the number of duplicates removed, the number of records screened and excluded at the title and abstract stage, the number of full texts assessed, and the rationale for exclusion during the eligibility evaluation. Upon completion of this comprehensive screening process, eighteen studies were deemed suitable and incorporated into the final qualitative synthesis. Within the context of assisted reproductive technologies, these studies together contribute to the current body of knowledge regarding the genetic and molecular functions of *TUBB8*, *KIF11*, and *CKAP5* in human oocyte maturation, spindle assembly, and early embryonic development.

### 2.5. Information Withdrawal

Following the inclusion of the 18 qualifying studies, a comprehensive data extraction process was conducted to ensure consistency, repeatability, and methodological rigor. Two reviewers independently extracted all relevant data using a uniform, pre-tested matrix developed in Microsoft Excel. The matrix was designed to encompass information across molecular, phenotypic, methodological, and clinical domains. A pilot extraction, first conducted on five papers, sought to verify uniformity in scope and interpretation. Consensus facilitated the resolution of discrepancies among reviewers; in instances of persisting doubt, a third reviewer served as an adjudicator.

The extracted fields included the following: (1) the name of the first author and year of publication to establish the chronological distribution of findings; (2) the gene(s) under investigation—namely *TUBB8*, *KIF11*, or *CKAP5*—given their known roles in microtubule dynamics and spindle regulation during human oogenesis; (3) the study design, categorized as case report, case series, cohort study, animal model, or experimental in vitro investigation; (4) the biological sample analyzed (e.g., oocytes, zygotes, embryos, HeLa cells); (5) the type of mutation identified, including missense, compound heterozygous, homozygous, frameshift, or nonsense variants, and their pathogenicity predictions; (6) the type of functional analysis conducted to validate mutation effects, such as immunofluorescence, Western blotting, HeLa cell transfection, 3D protein modeling, or in vitro maturation assays; and (7) whether the study reported outcomes related to in vitro fertilization (IVF) or intracytoplasmic sperm injection (ICSI).

Table 3 encapsulates the essential data fields derived from each of the 18 studies included. The table outlines the author and publication year, the gene(s) studied, the research design, the type of biological sample examined, the characteristics of the identified mutation(s), the molecular or functional methodology employed for validation, and the assessment of IVF outcomes. This organized matrix formed the foundation for the ensuing qualitative synthesis of the molecular pathophysiology and clinical ramifications of each variety.

Table 3 presents a comprehensive summary of the acquired data, providing a relative overview of all eighteen studies. The table underpins the mechanistic and clinical synthesis presented in the Section 3 and Section 4 and reveals significant trends in the literature. Of the 18 studies examined, 15 indicate that *TUBB8* is the most extensively researched gene, as evidenced by a comprehensive analysis of Table 3. This predominance aligns with the gene’s only role in encoding a β-tubulin isotype specifically expressed in oocytes and early embryos, as well as its expression profile unique to primates. Numerous mutations identified in *TUBB8*—such as p.R320H, p.A54V, p.E108K, and p.Q134*—have consistently been associated with pronounced phenotypic consequences, including GV arrest, MI arrest, substantial polar body extrusion, or cleavage failure. This research technique, frequently corroborated by functional validation in HeLa cells, oocytes, or animal models, employed both Sanger sequencing and next-generation sequencing (NGS).

Conversely, although less frequently acknowledged, *KIF11* and *CKAP5* are emerging contributors to meiotic fidelity. Limited studies on these genes have focused on their roles in spindle bipolarization, chromosomal congression, and microtubule anchoring in the absence of classical centrosomes. The integration of confocal imaging with functional studies of human or porcine oocytes demonstrated that the inhibition or mutation of these genes leads to spindle collapse, loss of tubulin acetylation, and lack of chromosomal alignment—phenomena that clearly correspond to clinical observations in IVF contexts.

The study designs within the corpus of literature varied significantly. Initial research predominantly consisted of case reports and small case series; contemporary studies have incorporated molecular profiling of larger patient cohorts utilizing standard IVF methodologies. In vitro systems, specifically through HeLa cell transfection or mouse oocyte injection, provided mechanistic evidence for the detrimental effects of the identified changes. These models consistently exhibited aberrant spindle morphology, compromised microtubule integrity, and a decline in oocyte maturation capacity.

In numerous studies, a notable strength was the integration of functional analysis. Fourteen of the eighteen studies sought to empirically validate the molecular impacts of genetic variants beyond mere sequence identification. Cao et al. and Zhang et al. demonstrated that *TUBB8* mutations in HeLa cells and mouse oocytes result in elevated embryonic fragmentation rates, disrupt α/β-tubulin dimer formation, and hinder spindle assembly [25,33]. Wan et al. similarly found that downregulation of CDK1, in conjunction with *KIF11* inhibition in pig oocytes, abolished polar body extrusion and altered the cell cycle [29]. Significantly, 15 of 18 studies (83%) reported clinical outcomes associated with IVF or ICSI, hence facilitating the direct application of molecular findings to reproductive prognosis. The outcomes included rates of oocyte maturation, fertilization success, cleavage progression, blastocyst development, and, in rare instances, live birth [29]. Some studies identified abnormal zygotic pronucleation or early embryonic arrest, while others documented total cleavage failure in zygotes derived from oocytes with pathogenic *TUBB8* mutations. Conversely, a few patients exhibiting milder variants, such as p.A313V, were shown to achieve pregnancy, highlighting the spectrum of phenotypic severity. The systematic extraction of genetic, clinical, and methodological attributes from all 18 studies provides a detailed understanding of how mutations in *TUBB8*, *KIF11*, and *CKAP5* restrict oocyte developmental competence. In women experiencing unexplained IVF failure, this matrix aids in elucidating the molecular pathways discussed in Section 3 and underscores the therapeutic significance of early genetic screening and functional validation.

### 2.6. Assessment of Bias Risk

Five predetermined domains were employed to assess the internal validity, methodological transparency, and translational robustness of the studies, including the following: (i) overall quality of study design, (ii) adequacy of sample size, (iii) application of functional or mechanistic validation, (iv) incorporation of IVF or ICSI outcomes, and (v) a comprehensive evaluation of overall risk of bias. This paradigm was adapted from previous molecular assessments in reproductive genetics, utilizing the unique attributes of investigations including both clinical and experimental domains.

One can assess the design quality by examining the methodological rigor, reporting clarity, control of confounding variables, and relevance of the experimental system. High-quality studies encompassed those with well-defined patient populations, clearly articulated genetic screening methodologies, and the inclusion of replication or control procedures. For example, low-risk, high-quality studies were identified, such as Feng et al. (2016) and Lu et al. (2021), which provided multi-patient evidence with unequivocal molecular validation and distinct molecular confirmation [26,32]. In contrast, certain studies lacking a structured experimental design or depending solely on individual case reports (e.g., Li et al., 2025; Yuan et al., 2021) were classified as moderate- or high-risk in this area [28,38].

An additional significant factor was the sample size. Despite the inherent rarity of TUBB8-associated oocyte maturation cessation and other genetic infertility disorders, variations in cohort size contribute to differing levels of evidentiary robustness. Limited sample sizes, namely less than three patients or oocytes, impose considerable constraints on generalizability and replicability. For example, numerous preliminary investigations, like Lanuza-López et al. (2020), concentrated on one to two patients and lacked external replication [31]. In contrast, Hu et al. (2023) and Wu et al. (2024) documented medium-to-large sample numbers (>10 patients or >100 oocytes), enhancing our confidence in the reported symptoms and mechanistic elucidations [36,37].

Low-bias studies were differentiated from the others by the use of functional validation trials. The experiments encompassed in vitro maturation (IVM) assays in murine or porcine oocytes, protein-level analyses (Western blot), microtubule visualization (α-tubulin immunofluorescence), and transfection studies comparing mutant and wild-type HeLa cells. Cao et al. (2021), Wan et al. (2018), and Hu et al. (2023) provided particularly robust mechanistic evidence, and fourteen studies incorporated at least one form of functional validation [25,29,36]. These findings validated that mutant variants of TUBB8, KIF11, or CKAP5 modify spindle morphology, disrupt microtubule networks, or restrict polar body extrusion, thereby corroborating the pathogenicity of the identified mutations [25,29,36]. Conversely, four investigations reported sequencing results lacking corroborative evidence on subsequent functional disturbances, rendering them more susceptible to bias in this domain.

The evaluation of clinical outcomes, including oocyte maturation, fertilization rates, cleavage progression, and embryo development, was conducted for translational significance. Fifteen of the examined trials (83%) documented IVF or ICSI outcomes directly associated with the identified mutation, hence significantly enhancing their clinical interpretability. Research conducted by Feng et al. and Zhang et al. demonstrated both the molecular consequences of certain mutations and their phenotypic manifestations, including cleavage arrest, multi-pronuclei formation, and embryo fragmentation [8,33]. Although experimentally rigorous, studies such as Wu et al. (2023) were assessed as having poorer translational completeness because to their failure to correlate findings with human IVF data [37].

The overall risk of bias was assessed based on the aggregation of the aforementioned areas. Six studies were categorized as having a low risk of bias due to their robust methodological quality, demonstrated mutation effects, and integration of clinical outcomes. The current review’s evidentiary foundation primarily relies on this study, which also significantly informs the synthesis in Section 3. Eight studies were categorized as intermediate risk, typically due to insufficient clinical connection, limited sample size, or lack of in vitro functional data. Four studies, primarily early case reports, were classified as high risk due to methodological opacity, lack of reproducibility, or absence of subsequent phenotypic or clinical analysis.

Table 4 provides an overview of this assessment, along with the methodological guidelines and risk evaluations for each of the eighteen studies included. This structured evaluation not only contextualizes the robustness of the given molecular evidence but also directs the interpretation of genotype–phenotype relationships in the subsequent synthesis. The comprehensive corpus of research consistently demonstrates the detrimental impact of *TUBB8*, *KIF11*, and *CKAP5* mutations on human oocyte maturation and early embryonic development, regardless of variations in study design and scope. The substantial proportion of studies featuring functional validation and reporting on IVF outcomes underscores the translational advancement of the field and facilitates the incorporation of more molecular diagnostics into infertility treatment.

Table 4 delineates the risk of bias for each included study according to five principal domains: quality of study design, sample size, existence of functional validation, reporting of IVF or ICSI outcomes, and the overall assessed risk of bias. The classification demonstrates methodological rigor and translational thoroughness.

## 3. Results

Eighteen original research publications were incorporated into the final qualitative synthesis, adhering to the established inclusion and exclusion criteria outlined during the systematic screening procedure. These studies were chosen from an initial compilation of 467 records obtained from four primary databases—PubMed, Scopus, Web of Science, and Embase. Following the elimination of duplicates and the screening of titles and abstracts, 43 full-text articles were evaluated for eligibility. A total of 18 studies satisfied the inclusion criteria; 25 full texts were excluded due to insufficient molecular data, lack of clinical relevance, or absence of mutations in TUBB8, KIF11, or CKAP5.

The PRISMA 2020 flow diagram (Figure 1) clearly illustrates the entire study selection process, detailing the reasons for exclusion at the full-text level together with the counts of detected, screened, excluded, and included records.

The research, conducted from 2016 to 2025, employs various methodological tools, including human genetic case series, functional validation experiments in HeLa cells and animal oocytes, and transcriptome profiling. A limited number of studies examined *KIF11* or *CKAP5*, whereas the majority focused mostly on TUBB8 [16,17,18,19]. All research provided original data on gene variations, incorporating both molecular and reproductive outcome components, hence facilitating the establishment of genotype–phenotype correlations.

### 3.1. Molecular Landscape and Mechanisms of TUBB8 Variations

The β8-tubulin isotype, encoded by the TUBB8 gene, is predominantly expressed in human oocytes and preimplantation embryos and is exclusive to primates. Crucial components of the meiotic spindle, α/β-tubulin heterodimers, evolve into dynamic microtubules. TUBB8 exhibits reduced redundancy compared to other β-tubulin paralogs, so the oocyte relies significantly on its structural and functional integrity. The oocyte-specific expression of TUBB8 indicates that even monoallelic pathogenic mutations can significantly impair meiotic fidelity, often manifesting as primary infertility characterized by oocyte maturation arrest or early embryonic developmental failure.

A wide mutational spectrum was identified throughout the 15 investigations focusing on TUBB8. This encompasses around thirty distinct variations distributed across functionally significant regions of the β8-tubulin protein. Although nonsense mutations (e.g., p.Q134*) and frameshift variants (e.g., p.Arg306Serfs*21) were also recorded, these alterations were predominantly missense in character. Highlighting the diverse inheritance patterns, compound heterozygous configurations and de novo occurrences were observed. In particular, five residues—p.I4L, p.E108K, p.V255M, p.G98R, and p.A313V—were consistently modified across disparate families, suggesting potential mutational hotspots.

Pathogenic mutations are structurally clustered in regions critical for microtubule dynamics, including the GTP-binding site, the longitudinal intradimer interface, and the lateral interdimer contact zones. The folding of β-tubulin, its dimerization with α-tubulin, and its incorporation into the polymerizing microtubule lattice are contingent upon these regions. The p.R320H and p.F265V mutations obstruct heterodimer formation at the intradimer interface, while the p.E108K and p.A54V alterations interfere with GTP binding. Utilizing PyMol structural modeling, these differences induce steric conflicts, disrupt hydrogen bonding networks, and destabilize β-strand architecture, resulting in protein misfolding and subsequent proteasomal degradation.

Functionally mutated β8-tubulin proteins, when expressed in HeLa cells, mice oocytes, or human oocytes, exhibit dominant negative effects. Mutant *TUBB8* proteins are incorporated into the microtubule cytoskeleton but interfere with the spindle apparatus, as demonstrated by in vitro expression studies. Immunofluorescence labeling revealed disrupted spindle polarity, fractured or multipolar spindles, and abnormal polar body extrusion. These in vivo deficiencies led to embryonic fragmentation, fertilization failure, or arrest at the GV or MI stages. Cao et al. [25] demonstrated that p.A54V caused the abnormal extrusion of larger polar bodies, whereas p.R320H led to arrest at metaphase I with condensed but misaligned chromosomes. The compound heterozygous variants p.Q134*/p.F265V resulted in oocytes that progressed to MII but exhibited no cleavage.

*TUBB8* mutations at the molecular level disturb a sophisticated network of intracellular pathways converging upon the coordination of meiotic spindle assembly, microtubule dynamics, and cytoskeletal integrity in human oocytes. The spindle assembly pathway is among the main disturbed mechanisms. Stable α/β-tubulin dimers, the building blocks of meiotic microtubules, are formed by β8-tubulin, the protein product of *TUBB8*, heterodimerizing with α-tubulin. Variants in *TUBB8* disrupt this dimer formation especially those influencing the intradimer contact zones or the GTP-binding interface. This greatly reduces the nucleation and elongation of kinetochore microtubules. Human oocytes, which lack centrosomes and depend on acentrosomal mechanisms for spindle organization, especially suffer from this consequence. Under such a background, even small changes in tubulin polymerization weaken spindle bipolarity and raise susceptibility to meiotic arrest.

PTMs of β8-tubulin, which are fundamental in controlling microtubule dynamics and protein recruitment during meiosis, also constitute another important axis of disturbance. In healthy oocytes, acetylation and detyrosination of β-tubulin control their interaction with motor proteins such dynein and kinesins (including *KIF11*) as well as anchoring elements needed for spindle pole focusing. These PTMs have been demonstrated to be interfered with by mutations such as p.V255M, resulting in poor pole integrity, lost coordinated spindle movement, and lower motor complex recruitment. These results imply that *TUBB8* mutations not only affect mechanical spindle assembly but also disturb the molecular signaling milieu controlling spindle stability.

A third site of susceptibility is the GTPase cycle. The dynamic instability of microtubules—a process wherein tubulin polymers undergo fast cycles of growth and shrinkage, allowing proper spindle orientation and chromosome alignment—is dependent on the inherent GTP-binding site of the β8-tubulin monomer. Mutations including p.A54V and p.E108K either inside or near this GTP-binding domain cause reduced GTP hydrolysis. Reduced turnover at microtubule plus-ends, loss of microtubule plasticity, and persistent abnormal microtubule configurations follow from this loss of microtubule plasticity, finally leading to failure of spindle self-organization and activation of meiotic checkpoints.

Moreover, *TUBB8* mutations seem to interfere with oocyte proteostatic and translational machinery. Many investigations have shown that mutant β8-tubulin is unstable and rapidly breaks down probably because of misfolding or failure to incorporate into microtubules. Human oocytes, which must rely only on mother mRNA and protein stores and are transcriptionally silent during meiotic maturation, are especially vulnerable from this destruction. The lack of replenishment systems for β8-tubulin increases the molecular effects of the mutation and results in permanent spindle failure. This results in continuous SAC activation, a fundamental protection mechanism controlled by elements including MAD2 and BUB1, which arrests meiosis until appropriate kinetochore– microtubule attachments are reached. Within a *TUBB8* mutant scenario, SAC activation becomes chronic and finally stops meiotic development.

Clinically, the variety of phenotypic expressions seen in affected patients reflects the effect of these genetic abnormalities. With oocytes failing to resume meiosis and reach the MII stage, the most severe presentations are GV or MI arrest. Sometimes fertilization takes place, but the resulting embryos show either cleavage failure, arrest before the eight-cell stage, or multi-pronuclear (MPN) zygotes. Studies including polymorphisms like p.Q134*, p.F265V, or compound heterozygous combinations routinely show complete fertilization failure or developmental arrest by day three. Fascinatingly, a small proportion of patients with milder variants—such as p.A313V or p.T429M—have shown partial developmental competency, including blastocyst stage development and, in extremely rare occasions, successful implantation. These results, however, might show hypomorphic alleles with residual function instead of actual harmful mutations.

Extracted from the literature, the genotype–phenotype correlations in Table 3 highlight the harmful potential of both missense and truncating variations. Often resistant to any kind of ART intervention, patients carrying homozygous or compound heterozygous null variants have the most severe effects. Monoallelic missense mutations, on the other hand, exhibit varying expressivity and penetrance, most likely influenced by the position of the variation, the structural impact on the tubulin fold, and compensating molecular mechanisms.

Table 5 provides a comprehensive summary of the gene variants identified in the eighteen studies included in this systematic review. The table encompasses the study design, origin, studied gene(s), identified variations, associated oocyte and/or embryonic phenotypes (e.g., GV arrest, MI arrest, cleavage failure), and the reported impact on IVF or ICSI outcomes. These investigations underscore the critical functions of oocyte competence and spindle dynamics, revealing the clinical and molecular diversity of mutations in *TUBB8*, *KIF11*, and *CKAP5*.

These results in Table 5 confirm the translational significance of *TUBB8* in clinical infertility. Patients with inexplicable IVF failure, aberrant oocyte shape, or polar body anomalies should have thorough genetic counseling and thought given to tailored ART plans based on *TUBB8* variations. Future therapeutic treatments are further enabled by recent research investigating the microinjection of wild-type *TUBB8* cRNA into damaged oocytes showing promise in restoring spindle activity and rescuing early embryonic development in animal models.

### 3.2. Variations in KIF11 and CKAP5: Novel Meiotic Regulators

While the majority of research to date has focused on *TUBB8*, increasing genomic investigations of individuals with oocyte maturation arrest and early embryonic failure have revealed additional significant functions in meiotic spindle assembly. The genes *KIF11* and *CKAP5* have emerged as significant non-redundant components of the cytoskeletal structure of the human oocyte. Both are crucial for spindle integrity, bipolarity, and chromosomal congression; mutations in their coding sequences have been associated with severe reproductive problems, including metaphase I stoppage and cleavage failure. In the acentrosomal context of mammalian oocytes, these genes not only operate downstream of *TUBB8* but also participate in a molecular network that collaboratively assembles and regulates spindle activity.

*KIF 11* encodes Eg5, a tetrameric kinesin-related motor protein that produces plus-end-directed sliding forces along antiparallel microtubules. The preservation of spindle bipolarity and pole–pole separation relies on this activity. Eg5 is drawn to centrosomes in mitotic somatic cells, where it operates in conjunction with γ-tubulin and pericentriolar material. In contrast, oocytes rely on spindle architecture that is self-organized and derived from the microtubule-organizing center, and they do not possess centrosomes. *KIF11* assumes significantly greater importance in this context. Wan et al. [29] demonstrated through pig oocyte models that the monastrol-mediated suppression of *KIF11* results in complete failure of first polar body extrusion, distortion of spindle morphology, and downregulation of Cdc2 (CDK1), the principal regulator of M-phase progression. A significant decrease in α-tubulin acetylation associated these effects with *KIF11*, indicating that it may indirectly regulate tubulin PTMs, thus influencing spindle stiffness and motor protein recruitment [29].

Notably, recent single-cell transcriptome studies have demonstrated that *KIF11* expression diminishes with oocyte aging, indicating a relationship between age-associated spindle instability and kinesin-mediated dynamics. Pathogenic mutations in *KIF11*, especially those impacting its ATPase motor domain or stalk regions, likely impair its ability to crosslink microtubules and generate directional force, resulting in either static, collapsed spindles that cannot facilitate chromosome alignment or multipolar spindles. These anomalies in humans are associated with metaphase I arrest, misaligned kinetochores, and the generation of oocytes with multiple pronuclei (MPN), likely indicating errors in spindle-derived signals essential for appropriate cortical granule exocytosis and sperm chromatin decondensation.

*CKAP5*, often referred to as *TOGp* or *XMAP215*, functions as a microtubule polymerase and bundler, serving as a molecular platform for microtubule elongation at the plus-ends. The many HEAT repeat domains interact with α/β-tubulin dimers, facilitating their incorporation into nascent microtubules. In the unique acentrosomal environment of the oocyte, where microtubule nucleation transpires through non-canonical structures such as the recently identified huoMTOC, this activity is crucial. *CKAP5* was demonstrated using high-resolution 3D confocal imaging to dynamically translocate from the subcortical huoMTOC to kinetochores following nuclear envelope breakdown (NEBD), thereby orchestrating the initiation and stabilization of bipolar spindle formation.

Mutations in *CKAP5* are hypothesized to impair tubulin-binding ability or diminish its multimerization capability, hence altering spindle microtubule density and orientation. Either fragmented spindle assembly or monopolar collapse precludes adequate chromosomal congression. Additionally, *CKAP5* constitutes a molecular triangle that integrates microtubule growth with spatial regulation of spindle orientation by direct interactions with other crucial oocyte proteins such as *TACC3* and *TPX2*. Disruption of this axis can irreversibly halt oocyte maturation at MI or MII and induce SAC activation due to a failure in kinetochore tension.

Despite arising from distinct genetic origins, the functional consequences of *KIF11* or *CKAP5* dysfunction mirror those of *TUBB8* mutations. *KIF11* mutations impair force generation and dynamic microtubule activity, *TUBB8* mutations affect the spindle’s structural core, and CKAP5 mutations disrupt polymer formation and microtubule architecture. Investigations of co-expression indicate that these proteins converge at significant cytoskeletal milestones. In cells expressing both mutant *TUBB8* and faulty *CKAP5*, spindle formation is entirely abolished, thereby highlighting the synergistic failure in structural assembly and dynamic elongation.

Clinically, patients with *KIF11* or *CKAP5* mutations exhibit a phenotype consistent with early embryonic fragmentation, *MPN* development, or severe oocyte maturation block. Some patients may not achieve cleavage or blastocyst development despite normal follicular growth and substantial egg production, indicating that changes in the oocyte’s intrinsic competence rather than ovarian response may be responsible. Importantly, these patients frequently exhibit normal karyotypes and hormone profiles, making molecular diagnosis essential to uncover the underlying cytoskeletal abnormalities.

*KIF11* and *CKAP5* are essential components of diagnostic gene panels for patients experiencing recurrent IVF failure due to their roles in oocyte-specific spindle assembly. Furthermore, their functions indicate attractive treatment targets. In mouse and pig models, in vitro oocyte rescue studies utilizing microinjection of wild-type cRNAs or *CRISPR* base editing are ongoing. Future approaches to address spindle-associated infertility may involve the restoration of spindle function through synthetic mRNA supplementation or pharmacological alteration of acetylation status, such as the use of *HDAC6* inhibitors.

### 3.3. Molecular Convergence and Common Pathways in Oocyte Maturation Arrest

Successful oocyte maturation and developmental competency rely on a precisely regulated molecular network responsible for spindle assembly, chromosomal segregation, cytoskeletal stability, and checkpoint control. In mammalian oocytes, particularly in human oocytes lacking canonical centrosomes, this network primarily relies on specialized cytoskeletal regulators and *aMTOCs*. Although their pathogenic effects converge on the same subcellular target—a compromised meiotic spindle that does not progress through metaphase and maintain genomic integrity—mutations in *TUBB8*, *KIF11*, and *CKAP5* interfere with distinct molecular pathways.

The primate-specific β-tubulin isotype, *TUBB8*, encodes heterodimers composed of α-tubulin, facilitating polymerization into spindle microtubules. Its molecular role is both structural and dynamic, since it regulates microtubule growth and shrinkage through GTP binding and hydrolysis, resulting in conformational changes—a phenomenon termed “dynamic instability.” Mutations identified in the GTP-binding domain (e.g., p.A54V, p.E108K) alter the nucleotide exchange rate and diminish the efficacy of catastrophe and rescue cycles. This leads to either excessively stable or unstable microtubules, both of which are detrimental in the oocyte environment where rapid spindle reconfiguration is necessary. Additionally, PTMs such as detyrosination and acetylation influence *TUBB8’s* interaction with microtubule-associated proteins (MAPs) and motor proteins. Decreased acetylation, particularly at lysine 40 of α-tubulin (indirectly affected by structural modifications of β-tubulin), has been shown to hinder the recruitment of dynein and *KIF11* to the spindle, hence diminishing microtubule anchoring and force transmission.

*KIF11* (Eg5) is a plus-end-directed homotetrameric kinesin that operates by crosslinking and sliding antiparallel microtubules apart, a crucial process that initiates and sustains the bipolar spindle shape. Continuous movement along microtubules relies on ATPase activity; mutations in the motor domain impair both mobility and mechanical force production. In *aMTOCs*, *KIF11* interacts with Aurora A kinase (AURKA) and pericentrin-like proteins (PLPs), thereby linking motor force generation to *MTOC* polarization and maturation. Incomplete *aMTOC* fragmentation or ineffective pole separation caused by disruption of this interaction may result in monopolar or multipolar spindles. Furthermore, *KIF11* dysfunction is associated with a subsequent decrease in *CDK1*/cyclin B activity. The processes of *NEBD*, chromatin condensation, and SAC silencing rely on substrates for *CDK1* phosphorylation, which include lamin A/C, *CDC25*, and *APC*/C. *KIF11* mutations indirectly induce meiotic arrest during the GV or MI phases by diminishing CDK1 activity.

*CKAP5*, sometimes referred to as ch-TOG or *XMAP215*, possesses several *TOG* and HEAT-repeat domains that bind unpolymerized tubulin dimers and facilitate their incorporation at the plus-ends of microtubules. This activity enhances microtubule polymerization rates and antagonizes *KIF2C* (*MCAK*), a protein that induces catastrophe. *CKAP5* is drawn to the huoMTOC in human oocytes and subsequently relocates to kinetochores and spindle poles during nuclear envelope breakdown (NEBD). In conjunction with *TACC3* and *TPX2*, it constitutes the spindle-stabilizing complex that protects microtubules from depolymerization and provides a framework for other *MAPs*. Pathogenic mutations in *CKAP5* diminish the conformational flexibility of its TOG domains or tubulin binding affinity, hence impairing its polymerase activity. This reduces spindle microtubule density and coherence, resulting in spindle disintegration and failure of kinetochore tension. Such problems result in persistent SAC activation because *BUBR1*, *MAD2*, and *CDC20* fail to detach properly from kinetochores, hence inhibiting the initiation of anaphase.

Mutations in *TUBB8*, *KIF11*, and *CKAP5*, which affect several molecular mechanisms, all lead to the activation or dysregulation of the SAC. The SAC ensures that anaphase commences only when all kinetochores are under adequate tension and attachment. These mutations generate abnormal spindles that either misalign or detach kinetochores, thereby activating MPS1 kinase and facilitating the recruitment of *MAD1*/*MAD2*. This halts APC/C-mediated degradation of securin and cyclin B, hence maintaining the oocyte in metaphase arrest. Moreover, particularly in oocytes with *KIF11* mutations, persistent SAC signaling has been linked to elevated levels of ROS and mitochondrial dysfunction. The availability of ATP is contingent upon mitochondrial integrity; thus, its shortage exacerbates spindle collapse. Research demonstrates that oocytes deficient in *KIF11* have reduced mitochondrial membrane potential and elevated caspase activity, implying that prolonged arrest leads to follicular atresia and apoptotic activation.

Human oocytes possess a distinct characteristic: they depend on maternal mRNAs and proteins, as transcription ceases at the germinal vesicle stage. Mutant β8-tubulin proteins (*TUBB8* variants), frequently recognized as misfolded and designated for proteasomal degradation, diminish the available functional tubulin reservoir during a crucial phase when protein resynthesis is impracticable. Similarly, dysregulated *CKAP5* and *KIF11* transcripts may produce unstable proteins or undergo nonsense-mediated decay. This leads to a state of proteostasis breakdown that triggers the unfolded protein response (UPR) and undermines future cytoplasmic maturation. Alterations in *TUBB8*, *KIF11*, and *CKAP5* collectively indicate synthetic lethality, wherein concurrent dysfunctions in multiple spindle-related pathways result in complete embryonic cessation. Clinical studies of compound heterozygous mutations or dual-gene alterations in patients exhibiting severe symptoms, such as cleavage-stage embryo arrest or large perivitelline space oocytes, corroborate this finding. These characteristics indicate a simultaneous breakdown of the mechanical and regulatory components of spindle assembly.

## 4. Discussion

This systematic review synthesizes data from 18 original studies examining mutations in *TUBB8*, *KIF11*, and *CKAP5*, offering a comprehensive overview of the molecular framework governing human oocyte maturation arrest. The evidence indicates the essential dependence of human oocytes on the integrity of the meiotic spindle apparatus, a dynamic structure governed by precise cytoskeletal regulation, motor-driven force generation, and the translational control of stored maternal mRNAs. A diverse array of pathogenic variations, ranging from single-nucleotide missense mutations to compound heterozygous and truncating alleles, was identified in the included investigations, correlating with a spectrum of reproductive diseases such as GV and MI arrest.

*TUBB8* is the most frequently mutated gene; it encodes a β-tubulin isoform uniquely present in oocytes and early embryos. Mutations in *TUBB8* alter GTPase-dependent microtubule dynamics, disrupt spindle morphology, destabilize α/β-tubulin heterodimers, and trigger the SAC. Significantly, *TUBB8* mutations affect both the geometry of the meiotic spindle and critical biochemical signaling pathways like as tubulin acetylation, detyrosination, and proteostatic balance, which collectively determine the oocyte’s ability to resume meiosis and undergo fertilization. The complete meiotic barrier to partial cleavage ability indicates that the phenotypic diversity of *TUBB8* mutations reflects diverse expressivity based on the mutation’s location and its effect on post-translational regulation.

*KIF11* and *CKAP5*, albeit less precisely characterized, have emerged as equally vital parallel regulators of meiotic development. *KIF11* encodes Eg5, a homotetrameric kinesin motor protein responsible for spindle pole separation and the sliding of antiparallel microtubules. Mutations in *KIF11* lead to defective force generation and the establishment of monopolar or multipolar spindles, resulting in chromosome misalignment, inadequate kinetochore attachment, and persistent activation of the SAC. *CKAP5*, a potent microtubule polymerase and bundling factor, facilitates the dynamic elongation and stabilization of spindle microtubules through its TOG domain-mediated interaction with free tubulin dimers. *CKAP5* mutations induce spindle collapse and kinetochore disarray via diminishing polymerization and plus-end tracking activity. Despite functioning independently of *TUBB8*, these proteins converge at critical cytoskeletal interfaces, including the acentrosomal human oocyte microtubule-organizing center, where they facilitate the early establishment of spindle bipolarity.

The functional impact of these mutations has been validated across various experimental platforms, including HeLa cells, murine and porcine oocytes, and human in vitro maturation systems, revealing consistent anomalies in spindle morphology, chromosomal alignment, and polar body extrusion. Clinical features closely reflect these molecular abnormalities. Patients with deleterious mutations frequently exhibit substantial egg production yet experience fertilization failure, early cleavage arrest, and a lack of viable embryos. While these results are atypical, certain hypomorphic mutations, like as *TUBB8* p.*A313V*, may infrequently facilitate blastocyst growth or pregnancy.

It is essential to emphasize that the majority of the studies conducted were performed in China. This may be attributed to China’s leadership in reproductive genetics research; nevertheless, it also implies the existence of specific constraints. Diverse communities may exhibit significantly distinct genetic histories, allele frequencies, and the manifestation of certain pathogenic variants. This may complicate the application of the results to more diverse or multi-ethnic communities. Moreover, modifications in clinical protocols, laboratory methodologies, and IVF success metrics may influence the comprehension of genotype–phenotype correlations. Further study is required from individuals of diverse ethnicities and geographical regions to validate and expand upon the current molecular findings.

### 4.1. TUBB8: Clinical Correlations and Molecular Definitions

Mutations in the TUBB8 gene significantly impact the early stages of meiosis, thus diminishing female fertility, and are well-established as a principal genetic cause of egg maturation arrest. TUBB8, which encodes a β-tubulin isotype, is essential for the formation of the meiotic spindle and is uniquely expressed in human oocytes and early embryos [26,42]. The alignment, segregation, and proper maturation of oocytes rely on the integrity of the spindle structure; thus, TUBB8 mutations undermine the architecture essential for oocyte developmental competence.

Pathogenic TUBB8 mutations converge at the molecular level across several critical cellular pathways. Missense mutations in the GTP-binding domain (e.g., p.A54V, p.E108K) impair GTP hydrolysis kinetics, which is crucial for microtubule flexibility and dynamic instability [43,44].

Additional variants, such as p.V255M and p.A313V, affect the sites of post-translational modifications (acetylation/detyrosination), thereby modulating the recruitment of motor proteins (e.g., dynein, kinesin) and spindle anchoring [45]. Collectively, these molecular defects impair kinetochore–microtubule interactions and spindle pole integrity, leading to either suboptimal embryo quality or a complete cessation of maturation.

Clinically, these molecular anomalies indicate their severity. In a thorough cohort examination of 89 women with oocyte maturation anomalies, Ebru et al. (2023) identified TUBB8 mutations in 10.1% of the cases. Seven of these mutations were novel, hence expanding the mutational spectrum [46]. No enduring pregnancies or live births were documented, while sporadic instances of biochemical pregnancy occurred in carriers of the c.535G>A mutation, indicating a partial although insufficient restoration of oocyte competence [46]. A 2018 study by Xiang et al. demonstrated family inheritance and phenotype consistency in severe loss-of-function mutations, identifying a unique p.A352S mutation in two sisters, both exhibiting GV arrest and total infertility [42].

Functional investigations corroborate these clinical findings. Yao et al. (2022) confirmed that these mutations affect both expression levels and cytoskeletal architecture, reporting that HeLa cells transfected with p.C239W, p.R251Q, and p.G96R exhibited significant reductions in tubulin protein expression and disrupted microtubule networks [45]. Chen et al. (2017) identified a homozygous deletion of the whole TUBB8 gene in one instance and a de novo compound heterozygous mutation in another, resulting in significant meiotic defects [47]. These results underscore the significance of allele status and mutation type in delineating the phenotypic spectrum [47].

Not all TUBB8 mutations result in complete meiotic cessation. The p.A313V mutation found by Yao et al. (2022) may represent a hypomorphic or partially functioning allele associated with cleavage-stage embryos [45]. Chen et al. (2017) documented several embryo features from MI arrest to fertilized zygotes that did not progress beyond the 2-cell stage [47]. These differences underscore the significance of genotype–phenotype association research and suggest the presence of compensatory mechanisms or moderator genes.

Encouraging results have emerged in the realm of therapy. Jia et al. (2020) demonstrated a proof-of-principle for functional rescue by microinjecting wild-type TUBB8 cRNA into mutant oocytes, which partially restored spindle assembly and polar body extrusion [48]. The findings indicate that TUBB8 mutations may be targeted for future treatment strategies, including gene editing, mRNA supplementation approaches, and diagnostic markers [48].

The cumulative data from the eighteen studies in our tables and the ten additional papers indicates that TUBB8 is required for human oocyte maturation. Suboptimal results in ART, such as failure of egg extraction, unsuccessful fertilization, or early embryo arrest, are frequently associated with truncating, homozygous, or compound heterozygous variations [46,47]. Missense mutations exhibit considerable variety; certain alleles may permit partial maturation and fertilization while still resulting in suboptimal embryonic development. TUBB8 is identified as a critical element in oocyte biology through the integration of molecular analysis and clinical ART data, hence validating its incorporation into standard preconception genetic screening panels for women experiencing recurrent oocyte maturation issues or unexplained IVF failures [49].

### 4.2. KIF11: A Key Molecular Motor in Oocyte Maturation and Meiotic Spindle Assembly

Kinesin Family Member 11 (KIF11), also referred to as Eg5, is an essential homotetrameric motor protein that facilitates bipolar spindle assembly during cell division by moving towards the plus end [50]. Its established role in mitosis has recently been expanded to meiosis, where it regulates oocyte spindle morphogenesis and chromosomal alignment. Various experimental models reveal that alterations in KIF11 function result in meiotic arrest, spindle instability, and chromosomal misalignment—elements closely associated with oocyte maturation failure and aneuploidy in human IVF [51].

Wan et al. (2018) demonstrated in pig oocyte models that pharmacological inhibition of *KIF11* with small-molecule inhibitors resulted in the failure of first polar body extrusion, hence indicating a stop during metaphase I [29]. In conjunction with this block, microtubule dynamics were significantly impaired, tubulin acetylation was diminished, spindles were disorganized, and chromosomal alignment was jeopardized [29]. Additional evidence indicating that *KIF11* functions upstream in cell cycle regulation was derived from the diminished expression of *Cdc2*, a crucial element of the maturation-promoting factor (MPF) complex [52]. The molecular signature characterized by arrest at MI, spindle aberrancy, and diminished MPF activity closely resembles profiles observed in oocytes from IVF cycles that cease development beyond metaphase.

Mishina et al. (2025) conducted a detailed examination of the spatial impacts of diminished *KIF11* expression on meiotic spindle development utilizing a *KIF11* haploinsufficient mouse model [53]. The research indicated that incomplete bipolarity and asymmetric spindle poles result from a partial loss of *KIF11* function. Although chromosomes in the inner equator of the spindle were properly oriented, those at the peripheral exhibited a lack of biorientation. This spatial anisotropy reveals a mechanism by which subthreshold expression might facilitate partial maturation while still resulting in diminished chromosomal segregation and developmental competence, indicating the uneven requirement for *KIF11* along the spindle axis [53].

The morphological commonality identified in human oocytes with unresolved maturation arrest underscores the therapeutic significance of these findings. Our systematic evaluation (Table 5) indicates that, in the absence of identified *TUBB8* mutations, a subset of patients exhibited oocytes arrested at metaphase I, characterized by aberrant spindle topologies and the absence of polar body extrusion. While direct genetic alterations in *KIF11* have not been recorded in human IVF populations, it is plausible that regulatory anomalies or compound heterozygous interactions with microtubule-associated proteins, such as *CKAP5*, may elucidate these effects [54]. Furthermore, transcriptome investigations of arrested oocytes often reveal downregulation of kinesin family proteins, indicating potential *KIF11* dysfunction in subfertility [29,53].

*KIF11* traverses antiparallel microtubules, separating spindle poles through ATP hydrolysis at the molecular level [55]. Proteins such as *TPX2*, *NuMA*, and *Aurora* A kinase facilitate the anchoring and stabilization of the spindle apparatus, hence ensuring precise coordination with this function [56]. Mishina et al. (2025) demonstrate that the suppression or functional deficiency of *KIF11* leads to improper alignment of chromosomes at the metaphase plate, resulting in erroneous attachments between kinetochores and spindle microtubules, a known contributor to aneuploidy and developmental block in embryos [53].

Additionally, the activity of *KIF11* is influenced by post-translational modifications of tubulin that modify the motor protein’s affinity for microtubules: acetylation and detyrosination. Oxidative stress or aging oocytes often result in alterations; this may elucidate the age-associated decline in *KIF11*-mediated spindle integrity [29]. These processes may elucidate the increased incidence of aneuploidy and developmental arrest in oocytes derived from older patients undergoing IVF.

*KIF11* functions as a dynamic motor that regulates the spatial geometry of the spindle apparatus, whereas *TUBB8* mutations directly diminish the incorporation of tubulin isotypes into the spindle [57]. Consequently, these proteins functionally converge to ensure proper spindle architecture and chromosomal biorientation, although operating at distinct levels: structural rather than mechanical. This necessitates more investigation into the combined effects of *TUBB8* and *KIF11* dysregulation on oocyte maturation issues.

### 4.3. CKAP5: A Cytoskeletal Architect in the Stability of the Meiotic Spindle

Cytoskeleton-Associated Protein 5, a member of the *XMAP215* family, is encoded by the *CKAP5* gene and is an essential microtubule polymerase. This protein promotes tubulin polymerization and stabilizes microtubule plus-ends, ensuring proper microtubule dynamics throughout mitosis and meiosis in a non-redundant manner. CKAP5 is crucial for the formation and maintenance of the meiotic spindle in the unique context of the human oocyte, which lacks typical centrosomes [29,58]. The absence or dysfunction of CKAP5 results in significant spindle shape abnormalities, failure of polar body extrusion, and ultimately meiotic arrest—phenotypes commonly observed in IVF cycles with unexplained metaphase I arrest [47].

Recent investigations have highlighted the dynamic expression pattern of CKAP5 during oocyte maturation. Beginning at the germinal vesicle breakdown (GVBD) stage, CKAP5 levels rise, reach a zenith during metaphase, and thereafter diminish after the zygotic genome activation phase, as evidenced by Western blot and immunofluorescence analyses [58]. This expression profile aligns with its cytoskeletal function, particularly in the organization and stabilization of the meiotic spindle apparatus. Confocal imaging demonstrated that *CKAP5* localizes to spindle poles and the whole spindle microtubule array, underscoring its essential role in spindle architecture and chromosome alignment [6].

The aforementioned factors result in metaphase I arrest; the functional suppression of *CKAP5* in murine oocytes disrupts spindle bipolarity, impedes proper kinetochore–microtubule attachments, and induces chromosome misalignment [58]. Co-immunoprecipitation investigations have demonstrated that *CKAP5* interacts with clathrin heavy chain (CLTC), hence substantiating the notion that *CKAP5* orchestrates microtubule bundling and spindle pole integrity in conjunction with structural spindle proteins [6]. Furthermore, the lowering of *CKAP5* significantly enhances kinetochore–microtubule stability, resulting in hyperstable attachments that resist correction—an error-prone configuration that predisposes cells to aneuploidy and embryonic arrest [6].

The importance of *CKAP5* extends beyond its structural roles. The *TOG* (Tumor Overexpressed Gene) domains of *CKAP5* elucidate its ability to cross-link microtubules and actin filaments, so providing a crucial interface between the two cytoskeletal systems. *CKAP5*, as stated by Sabo et al. (2024), is templated by dynamically unstable microtubules and facilitates the formation of persistent actin bundles [59]. Asymmetrical cell division in oogenesis relies on the proper spatial orientation of the spindle; thus, this interaction may be essential for spindle positioning and oocyte polarity. The *TOG5* domain has been identified as directly interacting with F-actin, hence facilitating the coordinated movement of cytoskeletal components during oocyte maturation [60].

The data indicate that irregularities in *CKAP5* expression or function may contribute to some oocyte maturation failures in IVF patients lacking mutations in standard spindle genes like *TUBB8*. Recent investigations have focused on *TUBB8* as a principal genetic cause of oocyte maturation arrest [42,46], although clinical observations indicate that most patients exhibiting similar spindle abnormalities lack identifiable *TUBB8* mutations. Under these conditions, *CKAP5* downregulation or functional inactivation through post-translational modifications (e.g., phosphorylation, degradation, or mislocalization) may be responsible [61]. Regrettably, a significant gap exists in reproductive genetics, as no substantial clinical sequencing investigation has examined IVF populations specifically for *CKAP5* mutations [62].

Furthermore, the functional convergence of *CKAP5* with *KIF11* and *TUBB8* suggests its role within a broader cytoskeletal framework responsible for oocyte meiotic competency. *TUBB8* provides structural integrity to spindle microtubules, while *KIF11* ensures motor-driven spindle elongation and bipolarity [53]. *CKAP5* functions as a polymerization catalyst and links microtubules with actin filaments, thereby integrating structural, dynamic, and positional control of the oocyte spindle. Dependable chromosomal segregation relies on this tripartite connection. All three may exhibit dysregulation manifesting as analogous clinical symptoms: myocardial infarction arrest, abnormal polar body extrusion, suboptimal fertilization, or premature embryonic developmental failure [26,45].

*CKAP5* has finally been associated with oocyte aging. According to Hu et al. (2025), aged oocytes exhibited diminished *CKAP5* levels, aberrant spindle formation, and increased aneuploidy [63]. This outcome indicates a mechanistic connection facilitated by *CKAP5* between ovarian aging and spindle instability, potentially offering a biological target for pharmacological interventions or rejuvenation therapies in IVF procedures for older mothers [63].

### 4.4. Prospective Directions and Integration with Molecular Pathophysiology

The incorporation of *TUBB8*, *KIF11*, and *CKAP5* into oocyte molecular biology highlights a meticulously regulated system in which cytoskeletal dynamics, spindle assembly, and chromosomal segregation intersect to establish ovarian competence and facilitate successful embryonic development [7]. Mutations in these genes reveal critical meiotic development checkpoints and highlight the molecular vulnerability of oogenesis.

*TUBB8*, a β-tubulin isotype only expressed in oocytes, fundamentally underpins this system and regulates spindle organization through microtubule polymerization and post-translational modifications. Loss-of-function mutations in *TUBB8*, such as p.V255M and p.A352S, impair acetylation and destabilize spindle poles, resulting in chromosomal misalignment and meiotic arrest [42,45]. Chen et al. (2017) identified structural deletions in *TUBB8*, further emphasizing the essential role of this gene in maintaining cytoskeletal integrity during metaphase I [47]. Findings from Ebru et al. (2023) reveal novel *TUBB8* polymorphisms that result in GV and MI arrest, as well as cleavage failure, hence reinforcing the association between phenotype and genotype [46].

*KIF11* is crucial for the elongation and polarization of the meiotic spindle, in addition to tubulin. *KIF11* crosslinks antiparallel microtubules and ensures chromosomal biorientation, functioning as a plus-end-directed homotetrameric kinesin [53]. The reduction of Cdc2 expression and the disruption of tubulin acetylation resulting from functional ablation in porcine oocytes corroborate its association with cell cycle regulation and microtubule stabilization [29]. Recent murine models indicate that haploinsufficiency disrupts outer equatorial chromosome alignment, leading to asymmetric segregation, hence suggesting regionally distinct biorientation requirements for *KIF11* [53].

In addition to the microtubule-centric perspective, *CKAP5*—also referred to as XMAP215 or ch-TOG—introduces a new dimension through its ability to synchronize microtubule polymerization with interactions with actin networks [59]. *CKAP5* interacts with clathrin heavy chain to facilitate spindle stability and localizes to the meiotic spindle [58]. Depletion of this factor, as demonstrated in rodent models and cancer cell systems, results in disrupted kinetochore–microtubule interactions and impaired embryonic development. Additional evidence of *CKAP5’s* unique ability to facilitate continuous actin bundling through microtubule templating is provided by Sabo et al. (2023) and Cammarata et al. (2024), thereby implicating this protein in the cytoskeletal interactions essential for spindle positioning [59,60].

Collectively, these findings demonstrate a tightly interconnected molecular network wherein scaffold protein interactions, tubulin dynamics, and kinesin-mediated force generation converge to regulate meiosis. Mutations or dysregulation at any point in this network, whether structural (*TUBB8*), motor (*KIF11*), or polymerase (*CKAP5*), can result in arrest phenotypes that are phenotypically identical yet mechanically distinct [2]. The elucidation of molecular pathways involving *TUBB8*, *KIF11*, and *CKAP5* has transformative potential in the diagnosis and treatment of infertility, particularly in cases with unresolved oocyte maturation arrest and early embryonic developmental failure. Traditional morphological assessments of oocytes exhibit limited predictive capability regarding maturation and fertilization outcomes. The integration of these genes into comprehensive NGS gene panels for reproductive genetics could facilitate the early identification of women at risk for meiotic arrest syndromes. *TUBB8* variants, ranging from null mutations to hypomorphic missense alterations such as p.A313V, may exhibit diverse meiotic behaviors and differing clinical prognoses [45,47].

The presence of hypomorphic variations, which produce partially functional proteins, facilitates the possibility of therapeutic intervention. In vitro, partial restoration of spindle formation and chromosomal alignment has been demonstrated by experimental methods, including cRNA microinjection of wild-type *TUBB8* mRNA into mutant oocytes [48]. Additionally, small-molecule chaperones or microtubule-stabilizing agents are being examined for their potential role in facilitating spindle formation in *TUBB8*-deficient oocytes, akin to chemical rescue strategies employed in neurodegenerative disorders characterized by microtubule dysfunction [64]. While these procedures remain exploratory, they may evolve into adjunctive ART treatments, particularly for patients possessing specific genotypes with residual functional capacity.

Future research should prioritize non-invasive oocyte quality evaluation, particularly by live imaging of meiotic spindle dynamics utilizing advanced microscopy techniques such as second harmonic generation (SHG) imaging or polarized light microscopy (PolScope). These tools facilitate functional phenotyping for potential genetic anomalies by observing spindle birefringence and dynamics while preserving oocyte integrity [42,47]. Parallel proteomic investigations of spindle-associated proteins, employing mass spectrometry and proximity tagging, may identify novel interactors or altered post-translational modifications in mutant oocytes, thereby enhancing molecular diagnostics.

Comprehensive functional rescue studies in patient-derived oocytes or induced pluripotent stem cell (iPSC)-derived oocyte-like cells could confirm the toxicity of specific mutations and investigate personalized rescue methodologies. High-resolution mapping of transcriptional disruptions associated with specific mutations in *TUBB8*, *KIF11*, or *CKAP5* can be achieved through the application of single-cell RNA sequencing (scRNA-seq) in halted or dysmorphic oocytes.

In experimental models such as mouse oocytes or human iPSC-derived germ cells, CRISpen-based genome editing, particularly CRISpen/Cas9 homology-directed repair (HDR), may be employed to correct pathogenic mutations or create isogenic mutant lines for functional assessments. This would provide definitive proof-of-concept for gene repair and facilitate the analysis of interaction networks, uncovering synthetic lethal vulnerabilities or compensatory mechanisms that may be exploited therapeutically.

The trajectory of reproductive medicine is likely to shift towards a precision medicine model, wherein extensive molecular diagnoses informed by genes such as *TUBB8*, *KIF11*, and *CKAP5* will dictate customized therapeutic regimens. The molecular correction of spindle anomalies, enhanced selection of viable oocytes, and the fabrication of functional gametes will be pivotal in shaping the future of infertility treatments.

## 5. Conclusions

This thorough study presents new insights into the molecular mechanisms of infertility in women with unresolved oocyte arrest syndromes, highlighting the essential roles of *TUBB8*, *KIF11*, and *CKAP5* in oocyte maturation and early embryonic development. Data synthesis from the studies 18 included and 10 additional high-impact publications indicates that mutations in these genes disrupt microtubule assembly and spindle integrity, resulting in severe clinical phenotypes such as metaphase I arrest, fertilization failure, and developmental arrest at the cleavage stage.

*TUBB8* mutations are the most often identified in clinical populations and are associated with various oocyte maturation anomalies, ranging from early embryo fragmentation to complete meiotic arrest. Novel mutations like as p.A313V, p.R251Q, and p.C239W have been shown to disrupt microtubule organization or destabilize the mitotic spindle, resulting in dominant-negative effects [45,46]. *KIF11* and *CKAP5*, two critical regulators of spindle dynamics and chromosomal segregation, were associated with altered tubulin acetylation, spindle pole formation, and the fidelity of kinetochore–microtubule attachment [6,29].

Molecular defects in several genes indicate a uniform cytoskeletal pathophysiology associated with multiple forms of oocyte maturation failure. These results underscore the necessity of incorporating high-resolution molecular diagnostics, including functional tests and next-generation sequencing panels, into clinical practice. Furthermore, experimental therapies offer intriguing avenues for therapeutic advancement, including cRNA injections, chemical chaperones, enhanced imaging techniques, and gene-editing systems.

*TUBB8*, *KIF11*, and *CKAP5* serve as molecular guardians of oocyte competence. Their instability indicates a significant vulnerability in the reproductive pathway, necessitating both continued research funding and clinical focus. Precision medicine methodologies in assisted reproduction offer enhanced diagnostic accuracy, refined patient stratification, and the development of novel therapies for women experiencing idiopathic infertility as our understanding of these biological mechanisms advances.

## 6. Clinical Recommendations and Implications

The personalization of infertility care significantly improves with the incorporation of *TUBB8*, *KIF11*, and *CKAP5* into the diagnostic framework of assisted reproduction. This evaluation yields multiple clinical suggestions for practice and future research based on the combined molecular data.

Women experiencing recurrent oocyte maturation failure, unexplained metaphase I arrest, or early embryonic arrest should consider targeted gene sequencing panels, including *TUBB8*, *KIF11*, *CKAP5*, and other microtubule-associated genes. Molecular screening may yield actionable diagnoses in a considerable proportion of patients, since research indicates pathogenic or possibly pathogenic mutations in TUBB8 in 10–32% of affected persons [45,46,47].

Secondly, with the application of functional annotations and in vitro studies, physicians should be capable of distinguishing among null, hypomorphic, and potentially benign variants. For example, hypomorphic variants such as *TUBB8* p.A313V or *KIF11* partial loss-of-function mutations may facilitate partial spindle development and oocyte maturation [53], thereby enhancing experimental protocols including cytoplasmic injection of wild-type transcripts, spindle-rescue compounds, or in vitro maturation protocols tailored to offset partial functionality.

Third, we recommend the incorporation of non-invasive technologies—such as high-resolution spindle imaging, time-lapse embryo monitoring, and proteomic profiling—to assess dynamic spindle competency and cytoskeletal integrity in real time. In IVF cycles where genetic testing is impractical or inconclusive, these procedures may prove highly beneficial.

Fourth, reproductive specialists should be aware of the specific mutations that have prognostic implications. Patients with compound heterozygous mutations in *TUBB8* are unlikely to benefit from conventional stimulation methods; early referral for oocyte donation may be more appropriate. In optimal laboratory conditions, patients with heterozygous missense mutations affecting *KIF11* or *CKAP5* may nonetheless achieve blastocyst formation [29,58].

From a translational research perspective, future efforts should focus on developing therapies that restore spindle integrity in vitro. Promising avenues that may transform the management of cytoskeletal infertility include gene therapy, RNA-based interventions, and tiny chemical chaperones targeting tubulin folding and stabilization.

The practical application of molecular diagnostics targeting spindle assembly genes has the potential to revolutionize current IVF standards. In previously untreatable instances of oocyte maturation arrest, customized oocyte evaluation, mutation-specific counseling, and targeted intervention strategies offer potential solutions.

## Figures and Tables

**Table 1:** Overview of Search Strategy and Database Output.

Database	Time Frame	Keywords Used	Filters Applied
**PubMed**	Jan 2010–Apr 2025	*TUBB8*, *KIF11*, *CKAP5*, oocyte maturation, spindle defects, IVF failure, genetic mutation, meiotic arrest	Humans; Full-text; English
**Scopus**	Jan 2010–Apr 2025	*TUBB8*, *KIF11*, *CKAP5*, oocyte maturation, spindle defects, IVF failure, genetic mutation, meiotic arrest	Humans; Article; English
**Web of Science**	Jan 2010–Apr 2025	*TUBB8*, *KIF11*, *CKAP5*, oocyte maturation, spindle defects, IVF failure, genetic mutation, meiotic arrest	English; Document type: Article; Human Studies
**Embase**	Jan 2010–Apr 2025	*TUBB8*, *KIF11*, *CKAP5*, oocyte maturation, spindle defects, IVF failure, genetic mutation, meiotic arrest	English; Human; Journal articles

**Table 2:** Inclusion and exclusion criteria for studies evaluating TUBB8, KIF11, and CKAP5 variants in oocyte maturation and IVF outcomes.

Category	Inclusion Criteria	Exclusion Criteria
**Study Type**	Original research articles (case reports, case series, cohort studies, experimental in vitro or in vivo studies)	Reviews, editorials, conference abstracts, duplicate studies
**Population**	Women undergoing IVF/ICSI with oocyte maturation arrest, fertilization failure, or early embryonic arrest	Studies not involving IVF-related infertility or unrelated to female reproductive outcomes
**Genetic Focus**	Studies investigating *TUBB8*, *KIF11*, or *CKAP5* mutations	Studies lacking specific genetic data on *TUBB8*, *KIF11*, or *CKAP5*
**Sample Type**	Human oocytes, embryos, granulosa cells, or cumulus cells	Non-human or irrelevant biological samples
**Outcomes**	Molecular validation, spindle morphology defects, embryonic cleavage failure	Absence of functional or molecular outcome data
**Language and Availability**	Full-text articles in English	Non-English publications or inaccessible full-texts

**Table 3:** Data extraction matrix for eighteen studies examining the role of *TUBB8*, *KIF11*, and *CKAP5* variants in oocyte and embryo dysfunction.

Author, Year	Gene Investigated	Study Design	Sample Type	Mutation Type	Functional Analysis	IVF Outcome Assessed
Cao et al., 2021 [25]	TUBB8	Case series	Oocytes, HeLa cells	Missense (p.R320H, p.A54V)	Microinjection, immunofluorescence	Yes
Feng et al., 2016 [8]	TUBB8	Family-based WES	Oocytes, embryos	Missense and homozygous deletions	Spindle assays, mouse oocytes	Yes
Feng et al., 2016 [26]	TUBB8	Family-based WES	Oocytes, embryos	7 missense mutations	Functional annotation	Yes
Huang et al., 2017 [27]	TUBB8	Case-control	Oocytes	Missense (p.V179M, p.R2M)	Sequencing only	Yes
Yuan et al., 2021 [28]	TUBB8	Case report	Zygotes	Homozygous (p.E108K)	Bioinformatics	Yes
Wan et al., 2018 [29]	KIF11	In vitro animal study	Porcine oocytes	Expression knockdown	Tubulin acetylation, Cdc2	No
Yang et al., 2020 [30]	TUBB8	Mutation screening	Oocytes, embryos	Missense, frameshift	Western blot, HeLa assay	Yes
Lanuza-López et al., 2020 [31]	TUBB8	Case series	Oocytes	Multiple (missense)	Sanger sequencing, IVF response	Yes
Lu et al., 2021 [32]	TUBB8	Cohort	Oocytes, embryos	5 novel, 5 known	In vitro and in silico	Yes
Zhang et al., 2023 [33]	TUBB8	Case report	Embryos	Compound heterozygous	Immunostaining, protein modeling	Yes
Wu et al., 2022 [34]	CKAP5	Imaging study	Human oocytes	Imaging-based spindle defects	Confocal imaging	No
Zhu et al., 2022 [35]	TUBB8	Retrospective cohort	Oocytes	Multiple missense variants	In vitro maturation & fertilization	Yes
Hu et al., 2023 [36]	TUBB8	Multicenter cohort	Oocytes, HeLa cells	5 novel, 5 known	Protein expression, tubulin disruption	Yes
Wu et al., 2023 [37]	KIF11, CKAP5, HAUS6	Imaging + genetics	Oocytes	Mutations in 3 genes	Confocal microscopy + gene identification	Yes
Li et al., 2025 [38]	TUBB8	Case series	Oocytes	3 novel variants	In vitro modeling	Yes
Qin et al., 2025 [39]	GTPBP4, CKAP5	Comparative transcriptomics	Prepubertal goat oocytes	Gene upregulation	RNAseq + in vitro maturation	No
Zhao et al., 2025 [40]	TUBB8, PATL2, WEE2	Exome sequencing	Embryos, oocytes	5 novel variants	HEK293T validation, protein modeling	Yes

**Table 4:** Risk of bias assessment across included studies based on study design, sample size, functional validation, and IVF outcome reporting.

Author, Year	Study Design Quality	Sample Size	Functional Validation	IVF Outcome Reporting	Overall Risk of Bias
Cao et al., 2021 [25]	Moderate	Small (2 families)	Yes	Yes	Low
Feng et al., 2016 [8]	High	Medium (9 families)	Yes	Yes	Low
Feng et al., 2016 [26]	High	Medium (24 families)	Yes	Yes	Low
Huang et al., 2017 [27]	Moderate	Medium (109 total)	No	Yes	Moderate
Yuan et al., 2021 [28]	Moderate	Small (transcriptomic, 12)	Yes (bioinformatics)	No	Moderate
Wan et al., 2018 [29]	Moderate	Animal model	Yes	No	Moderate
Yang et al., 2020 [30]	Moderate	Medium (115/200)	Yes	Yes	Moderate
Lanuza-López et al., 2020 [31]	Low	Small	No	Yes	High
Lu et al., 2021 [32]	High	Medium (5 families)	Yes	Yes	Low
Zhang et al., 2023 [33]	Moderate	Single case (2 families)	Yes	Yes	Moderate
Wu et al., 2022 [34]	Moderate	Large (>1000 oocytes)	Yes	No	Low
Zhu et al., 2022 [35]	High	Medium (28 patients)	Yes	Yes	Low
Hu et al., 2023 [36]	High	Medium (35 patients)	Yes	Yes	Low
Wu et al., 2023 [37]	High	Large (7 families)	Yes	Yes	Low
Li et al., 2025 [38]	Moderate	Medium (3 families)	Yes	Yes	Moderate
Qin et al., 2025 [39]	High	High-throughput animal model	Yes	No	Low
Zhao et al., 2025 [40]	High	Medium	Yes	Yes	Low

**Table 5:** Genetic findings, functional impact, and clinical outcomes from the included studies.

Year	Author	Type of Study	Inclusion Criteria	Country of Origin	Sample Size (Cases/Controls)	Spindle Related Gene Investigated	Results
2016	**Feng et al.** [8]	Retrospective cohort	Infertile women Oocyte maturation arrest diagnosed	China	Members of 9 families	TUBB8	7 heterozygous missense mutations were identified (S176L, V255M, R262W, T238M, I210V, T285P, N348S) 2 homozygous mutations were detected (27_A33del, 143Dfs*12)
2016	**Feng et al.** [26]	Retrospective cohort	Infertile women Oocyte maturation arrest diagnosed	China	Members of 24 families	TUBB8	7 mutations were identified (V229A, D417N, S176L, R262Q, M363T, R2K, M300I)
2017	**Huang et al.** [27]	Case-control study	Infertile woman Oocyte maturation arrest diagnosed Normal karyotype	China	109(9/100)	TUBB8	2 new heterozygous mutations were identified in affected individuals (c.535G > A (p.V179M), c.5G > T (p.R2M))
2018	**Yuan et al.** [41]	Case report	Infertile woman Complete cleavage failure Normal karyotype Normal ultrasound findings Normal laboratory status	China	n/a	TUBB8	1 homozygous mutation was identified in (c.322G > A (p.Glu108Lys))
2018	**Wan et al.** [29]	Animal study	Porcine oocytes	China	n/a	KIF11	Inhibition of *KIF11* activity was associated with the failure of the first polar body extrusion Inhibition of *KIF11* resulted in sequential disruption of the spindle assembly *KIF11* was involved with aging-induced spindle disorganization
2020	**Yang et al.** [30]	Case-control study	Women with repeated (≥2) failed IVF attempts due to underlying: oocyte maturation arrest fertilization failure MPN formation cleavage failure embryonic arrest mixed defects Women <40 years old Normal karyotype	China	315(115/200)	TUBB8	31 variants were identified (30 missense variants and 1 frameshift insertion variant), of which 15 were novel
2020	**Lanuza-López et al.** [31]	Case series	Infertile woman Normal karyotype Normal ultrasound findings Normal laboratory status Oocyte maturation arrest diagnosed	Mexico	n/a	TUBB8	3 missense variants were detected (NM_177987.2:c.735G>C (p.Gln245His), NM_177987.2:c.845G>C (p.Arg282Pro), NM_177987.2:c.763G>A (p.Val255Met) (rs782269374))
2021	**Lu et al.** [32]	Retrospective cohort	Infertile woman	China	Members of 5 families	TUBB8	5 missense variants were detected (c.208C > A(p.P70T),c.907 T > C(p.C303R),c.173G > A(p.R58K),NM_177987.2: c.326G> T(p.G109V), NM_177987.2: c.916C >T(p.R306C))
2021	**Cao et al.** [25]	Retrospective cohort	Infertile woman	China	Members of 2 families	TUBB8	2 missense variants were detected (p.A54V and p.R320H)
2021	**Yuan et al.** [28]	Retrospective cohort	Human oocytes arrested in Meiosis II by women that underwent ICSI	China	12	KIF11	Upregulated but uncertain of its role in oocyte ageing
2022	**Zhang et al.** [33]	Retrospective cohort	Infertile woman	China	Members of 2 families	TUBB8	1 heterozygous missense variant (c.1286C > T (p.Thr429Met)) and a novel compound heterozygous mutations c.915_916delCC (p.Arg306Serfs*21) and c.82C > T(p.His28Tyr)) were detected
2022	**Wu et al.** [34]	Retrospective cohort	Infertile woman ≥2 failed attempts of IVF/ICSI, characterized by oocyte maturation arrest <45 years old	China	1394	CKAP5	Down-regulation of CKAP5 along with other human oocyte microtubule organizing center components lead to impaired spindle microtubule nucleation and spindle assembly in human oocytes
2022	**Zhu et al.** [35]	Retrospective cohort	Infertile woman Oocyte maturation arrest diagnosed	China	28	TUBB8	3 heterozygous missense variants were detected (c.1302_1304dup p.Glu434dup, c.527C>T p.S176L, c.874C>A p.Q292K)
2023	**Hu et al.** [36]	Retrospective cohort	Infertile woman	China	35	TUBB8	9 heterozygous missense and 1 loss-of-function variants were detected 5 novel variants (c.37G > A, p.G13R; c.149 A > G, p.Y50C; c.407 C > T, p.T136I; c.793T > G, p.F265V; c.1096 A > G, p.T366A) and 5 previously reported variants (c.10 A > C, p.I4L; c.124 C > G, p.L42V; c.400 C > T, p.Q134*; c.763G > A, p.V255M; c.1045G > A, p.V349I)
2024	**Wu et al.** [37]	Retrospective cohort	Women who underwent ICSI for male factor infertility	China	Members of 7 families	KIF11	Mutations identified in the *KIF11* gene impaired spindle bipolarization leading to oocyte and embryonic developmental defects
2025	**Li et al.** [38]	Retrospective cohort	Infertile woman Prior IVF or ICSI failure	China	Members of 3 families	TUBB8	3 heterozygous missense variants were detected (c.A1207G, c.G917A, c.C568T)
2025	**Qin et al.** [39]	Animal study	Adult and prepubertal goat oocytes	China	n/a	CKAP5	Up-regulation of CKAP5 in the translatome during adult goat oocyte maturation but insufficient translation in prepubertal goats, possibly leading to alterations of nuclear maturation
2025	**Zhao et al.** [40]	Retrospective cohort	Infertile woman Prior IVF or ICSI failure due to embryonic developmental arrest	China	84	TUBB8	3 missense variants were detected 2 novel variants (c.604A>T, c.848C>A) and 1 previously reported (c.322G>A)

## Data Availability

Not applicable.
